# Successfully treatment by eribulin in visceral crisis: a case of lymphangitic carcinomatosis from metastatic breast cancer

**DOI:** 10.1186/s12885-018-4725-7

**Published:** 2018-08-20

**Authors:** Jean-David Fumet, Mark Wickre, Jean-Philippe Jacquot, Marie-Helene Bizollon, Adrien Melis, André Vanoli, Erika Viel

**Affiliations:** 1 Department of Oncology, Centre Georges François Leclerc, 1 rue Professeur Marion, 21000 Dijon, France; 2 Department of Radiology, University of Minnesota, 420 Delaware St. SE, Minneapolis, MN 55455 USA; 3 Department of Pathology, Ramsay Général de Santé, Hopital Privé Sainte Marie, 4 Allée de Saint-Jean-des-Vignes, 71100 Chalon-sur-Saône, France; 4 Department of Oncology, Ramsay Général de Santé, Hopital Privé Sainte Marie, 4 Allée de Saint-Jean-des-Vignes, 71100 Chalon-sur-Saône, France; 5 Department of Oncology, Chalon-sur-Saône and Institut de Cancérologie de Bourgogne, 4 allées St Jean des Vignes, 71100 Chalon sur Saône, France

**Keywords:** Breast cancer, Visceral crisis, Lymphangitic carcinomatosis, Eribulin

## Abstract

**Background:**

Metastatic breast cancer (MBC) rest an incurably disease associated with bad prognosis and a median overall survival of 23–31 months. There are several treatment options including chemotherapy and sometimes endocrine therapy. Currently, there is no standard treatment for patients with MBC who have already benefited from anthracyclines and taxanes therapy. Many drugs like capecitabine, eribulin, gemcitabine, vinorelbin and liposomal doxorubicin are conventionally used as monotherapy. One important complication from MBC is life threating visceral crisis that needs a fast-effective treatment.

**Case presentation:**

We report here a case of an evolution of metastatic breast cancer with lymphangitic carcinomatosis after taxane based chemotherapy and endocrine therapy. This 37-year-old woman was referred to our hospital with complaints of dyspnea and dry cough. There was clinical concern for visceral crisis and a chemotherapy with eribulin was initiated. Pulmonary lymphangitic carcinomatosis disappeared and the patient achieved a good partial response.

**Conclusion:**

We reported a case of rapid, positive treatment response using eribulin on metastatic breast cancer with visceral crisis and we could quoted others. Therefore, eribulin may be an appropriate chemotherapeutic option in instances requiring rapid symptom control.

## Background

Breast cancer is the most commonly diagnosed cancer and the first cause of cancer death in women in the world. Approximately 40% of patients with early breast cancer will develop metastatic disease [1]. Metastatic breast cancer (MBC) is still an incurable disease associated with poor prognosis and a median overall survival of 23–31 months [2].

In particular, management of patients with MBC associated with visceral metastasis continues to be a challenge of management. International guidelines recommend endocrine therapy for women with hormone receptor positive (HR+) MBC, except patients with visceral crisis, which is defined as the presence of lymphangitic lung metastases, bone marrow replacement, carcinomatous meningitis, or significant liver metastases [3, 4]. In these situations, it’s necessary to start a chemotherapy in order to obtain a rapid symptom control. In fact, patients receive multiple sequential lines of chemotherapy. Thus, the strategy of sequence of different drugs is important. Indeed, Bonotto et al. have shown that the response to the first line therapy seems to affect the response to the second line [5].

Currently, there is no standard treatment for patients with MBC who have already treated by anthracyclines and taxanes. Despite the lack of specific recommendations many drugs are commonly used as monotherapy like capecitabine, eribulin, gemcitabine, vinorelbine and liposomal doxorubicin. The choice will depend of toxicity, performans status and needed speed of treatment efficacy. Eribulin mesylate is a synthetic analog of halichondrin B. It is a natural product isolated from *Halichondria okadai*. Eribulin inhibits microtubule polymerization with an action mechanism that differs from those of taxane [6]. In the phase III EMBRACE study, eribulin improved overall survival (OS) compared with treatment of physician’s choice [7]. As a result of these findings, eribulin was approved for the treatment of patients with locally advanced or metastatic breast cancer who have progressed after at least two chemotherapeutic regimens, including anthracyclines and taxanes (in adjuvant or the metastatic). Furthermore, a recent study have shown that eribulin is also effective and well tolerated in taxane-refractory patients [8]. Lymphangitic carcinomatosis is a radiological and clinical entity accounting for approximately 8% of all cases of lung metastasis. It is characterized as the presence of tumoral cells in lymph vessels and lung interstitium, without lung parenchyma remodeling [9]. It prevents adequate blood gas exchanges, and therefore is often considered as a visceral crisis [3].

## Case presentation

In 2010, a 37 year-old black woman had mastectomy and homolateral axillary dissection for invasive carcinoma in her left breast. pTNM stage was pT3 multicentric (53 mm diameter for the biggest) pN2 (seven positive lymph nodes) and no metastasis. Proliferation index was high (Ki-67 = 40%). Immunohistochemistry showed 90% of ER positivity and 60% of PR positivity. No overexpression of HER2 receptors has been found. According to multidisciplinary concertation, the patient was treated with adjuvant chemotherapy with six courses of FEC 100 (LV5FU 500 mg/m^2^, epirubicin 100 mg/m^2^, cyclophosphamide 500 mg/m^2^) every 3 weeks. Thereafter, she was treated by tamoxifen, at a dosage of 20 mg/day, and triptorelin (agonist analog of luteinizing hormone releasing hormone) for 4 years, until February 2014. In February 2014, the clinical exam reported a skin relapse in place of mastectomy scar. A CT scan showed multiple and bilateral pulmonary lesions and left pleural effusion. Chemotherapy with paclitaxel 80 mg/m^2^ and bevacizumab 10 mg/kg was initiated. Follow up imaging showed a positive partial response, so maintenance with fulvestrant and bevacizumab was initiated in August, 2014.

In February 2016, due to further progression in lungs and multiple bones sites, she was treated with exemestane 25 mg and everolimus 10 mg with an initial partial response.

**Fig. 1:**
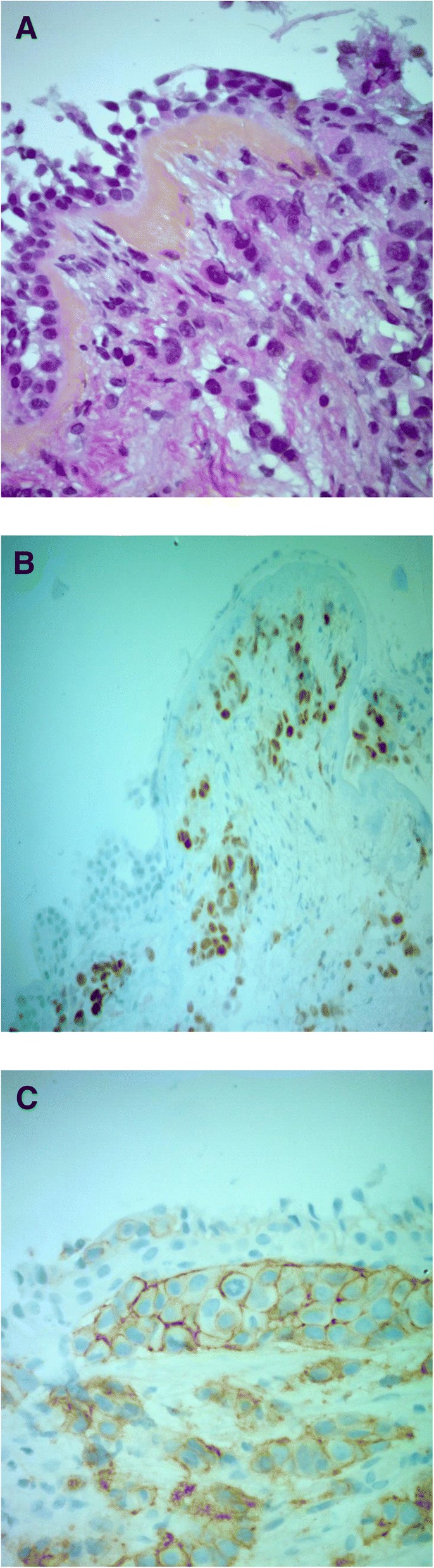
The bronchoalveolar lavage fluid showed many adenocarcinoma cells. **a** HPS × 200. **b** Immunohistochemistry (× 200) was positive for estrogen receptor. **c** Immunohistochemistry (× 400) was 2+ for HER2 (FISH negative)

In October 2016, she reported a dyspnea with dry cough. Left pleural effusion and non-specific infiltration were observed on the chest x-ray. We evocated first a mTOR inhibitor-associated non-infectious pneumonitis [10]. According to recommendations for patients with adverse events grade 3, everolimus was interrupted and corticosteroids administered. There was only a slight clinical improvement. The patient was submitted to bronchoscopy which shown a diffuse infiltration of lymphangitic appearance of the superior left trunk. The bronchoalveolar lavage fluid was negative for bacteria, acid-fast bacilli, and fungi. However, many adenocarcinoma cells were observed (Fig. 1).

Therefore, we concluded there was disease progression leading to visceral crisis and eribulin was started on 11.17.2016.

**Fig. 2:**
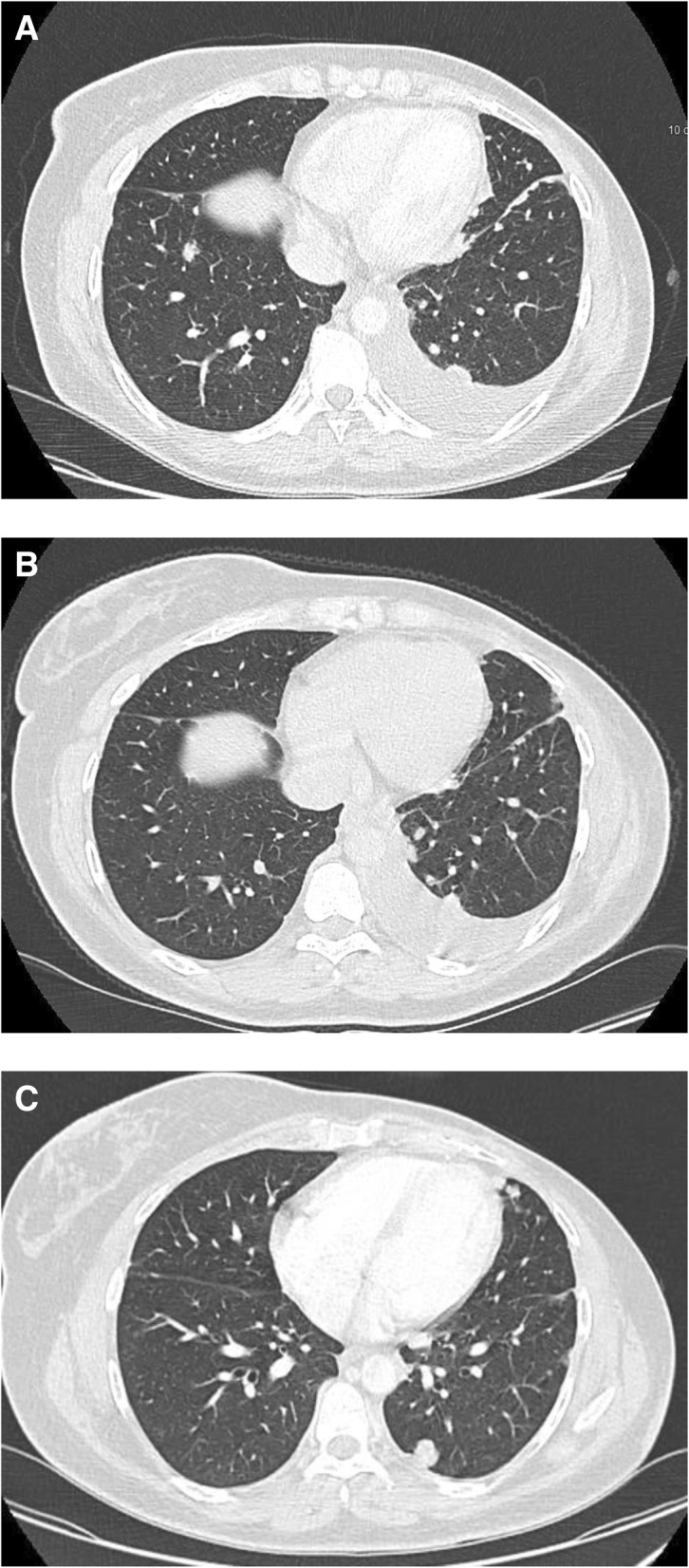
**a** CT scan showed irregular interlobular septal thickening and micronodular opacities. The infiltration and diffuse nodules distributed throughout the lymphatic vessels evocated lymphangitic carcinomatosis. **b** CT scan after the fourth cycle of the chemotherapy showed disappearance of micronodular invasion and a reduction of pulmonary nodules. **c** CT scan after 6 months with eribulin treatment

12.5.2016, a baseline CT scan was performed and revealed infiltration and diffuse nodules distributed throughout the lymphatic vessels confirming lymphangitic carcinomatosis (Fig. 2a). After only one course of the therapy, that is to say 14 days, a remarkable clinical response on the breathness and cough was noted.

After four courses of eribulin, a CT scan was performed and showed a significant reduction of pulmonary lesions and previously identified micronodules had disappeared (Fig. 2b). CT scan at 6 months confirmed radiological benefit (Fig. 2c).

Overall, this patient benefited of 8,5 months of eribulin with a significant clinical benefit. In August 2017, CT scan showed a major progression disease with several lesions in lung, hepatic and bones. She started a new therapeutic regimen by fluorouracil and vinorelbin with a satisfying efficacy on all target lesions. Progression free survival was 7 months. In March, 2018, the patient had a severe asthenia, dyspnea and diffuse bone pain. Chemotherapy was stopped and she benefited of best supportive care. Death occurred at hospital on 04.24.2018 with an overall survival since diagnostic of 50 months.

## Discussion and conclusions

Lymphangitic carcinomatosis is commonly characterized by dyspnea and a nonproductive cough. Chest radiographs appear non-contributory for 30–50% of patients with histologically confirmed disease [11]. Lymphangitic carcinomatosis results of the initial haematogenous spread of tumour to the lungs, with malignant invasion through the vessel wall into the pulmonary interstitium and lymphatics. In histology, there is a thickening of the supporting structures of the lung parenchyma and interstitium by tumor. This may be responsible for lymphatic obstruction. The presence of cancer cells inside of the lymphatic vessels may cause compression of the bronchioles and alveoli leading respiratory symptoms. This phenomenon increases the resistance of flow in the conducting airways with resistance to oxygen therapy. Furthermore, these symptoms can lead to pulmonary hypertension or pulmonary emboli in some cases.

Therefore, we can consider it as a visceral crisis in accordance with the Advanced Breast Cancer (ABC3) definition of severe organ dysfunction assessed by symptoms and important visceral compromise leading to a clinical indication for a more rapidly efficient therapy [4]. It is difficult to control lymphangitic carcinomatosis with chemotherapy and the prognosis is generally poor with a 50% mortality at 3 months [12]. Despite new anticancer strategies developed in recent years, there has been no effective strategy to treat lymphangitis carcinomatosis. To our knowledge, there is no data about efficacy of breast cancer therapy on lymphangitic spread. Platinum-based chemotherapy has obtained transient response in some patients with lung cancer [13]. Indeed, a phase 2 trial assessed and showed the efficacy of platinium-based therapy as monotherapy in pretreated patients who have advanced non-small-cell lung cancer. It suggests a good penetration in lung site [14].

In EMBRACE trial [7], objective response rate of eribulin was 12 and 5% with other treatments (physician’s choice). It suggests that eribulin may have the better response rate which is the major goal in visceral crisis. There has previously been no description of such rapid and effective treatment of visceral crisis with eribulin. In our case, eribulin’s PFS was 8,5 months. This result corresponds to the expected PFS (median PFS was 13,5 months in EMBRACE trial [7] and 3,1 months in retrospective data [8]. The most important point is the rapidity of response of eribulin in case of symptomatic disease.

In our local experience, we have identified two others cases with rapidly efficacious of eribulin on metastatic breast cancer. The first, a 38 years old young woman who was treated with many lines of chemotherapy for a metastatic breast cancer RH+ HER2- with hepatic and bones metastasis. In this patient, we also observed a major positive response because of eribulin treatment. Hepatomegaly decreased of more than 50%, ASAT 39 vs 116, ALAT 23 vs 100. Both CAE and CA15.3 were markedly decreased at 291 ng/ml vs 2917 ng/ml and 19 ng/ml vs 29.9 ng/ml respectively in only 14 days. A second patient was a 54 years old woman who was diagnosed with metastatic breast cancer. After numerous treatments, thrombopenia revealed a medullary bone marrow invasion. It was confirmed by bone marrow biopsy. After one course of eribulin, we have noted an increase of platelets to 40G/L to 100G/L in 14 days.

In conclusion, lymphangitic carcinomatosis should be considered as a visceral crisis secondary to the presence of organ dysfunction and chemotherapy is the therapy of choice. To our knowledge, few cases of metastatic lymphangitic carcinomatosis have been reported and there have no description about the effect of eribulin on this metastatic site.

Our observations suggest that treatment with eribulin can be rapidly effective for these patients and may be a good option in the emergency clinical situation with a visceral crisis. Further clinical studies are warranted to confirm this clinical effect.

## Abbreviations

ALAT: Alanine Aminotransferase
ASAT: Aspartate Aminotransferase
CEA: Carcinoembryonic antigen
ER: Estrogen Receptor
MBC: Metastatic Breast Cancer
PFS: Progression-free Survival
PR: Progesterone Receptor
